# Strain Relaxation of InAs Quantum Dots on Misoriented InAlAs(111) Metamorphic Substrates

**DOI:** 10.3390/nano12203571

**Published:** 2022-10-12

**Authors:** Artur Tuktamyshev, Stefano Vichi, Federico Guido Cesura, Alexey Fedorov, Giuseppe Carminati, Davide Lambardi, Jacopo Pedrini, Elisa Vitiello, Fabio Pezzoli, Sergio Bietti, Stefano Sanguinetti

**Affiliations:** 1 Isituto Nazionale di Fisica Nucleare, Sezione di Milano-Bicocca, 20100 Milano, Italy; 2 Department of Materials Science, University of Milano-Bicocca, 20100 Milano, Italy; 3 Centro Nazionale delle Ricerche, Istituto di Fotonica e Nanotecnologie, 20100 Milano, Italy

**Keywords:** droplet epitaxy, quantum dot, metamorphic buffer layer, strain relaxation, III–V semiconductors

## Abstract

We investigate in detail the role of strain relaxation and capping overgrowth in the self-assembly of InAs quantum dots by droplet epitaxy. InAs quantum dots were realized on an In${}_{0.6}$Al${}_{0.4}$As metamorphic buffer layer grown on a GaAs(111)A misoriented substrate. The comparison between the quantum electronic calculations of the optical transitions and the emission properties of the quantum dots highlights the presence of a strong quenching of the emission from larger quantum dots. Detailed analysis of the surface morphology during the capping procedure show the presence of a critical size over which the quantum dots are plastically relaxed.

## 1. Introduction

The self-assembled quantum dots (QDs) of compound semiconductors are promising candidates for the realization of on-demand entangled photon emitters that are fundamental components of future quantum communication networks [1,2,3,4,5]. The generation of entangled photon pairs requires highly symmetrical QDs to contrast the anisotropy-induced fine structure splitting (FSS) of excitonic states [6,7]. The C${}_{3v}$ symmetry of {111} surfaces renders them an ideal substrate for the formation of laterally symmetric QDs with naturally low FSS [3,8]. Single and entangled photon emitters operating at the telecom-wavelength bands (C-Band at ≈1.55 μm, and O-Band at ≈1.3 μm) for quantum key distribution over long distances emerged as major devices for quantum information technologies. Such long-emission wavelengths require the use of InA-based QDs grown on InP substrates or via the use of an InGa(Al)As metamorphic buffer layer (MMBL) [9,10,11,12,13,14,15,16,17]. In this regard, (111)-oriented substrates have a further advantage, as high-quality lattice-relaxed MMBLs can be formed by strained epilayers, permitted by the formation of misfit dislocations at the interface starting directly from the initial stages of the growth [18]. The fast relaxation of the strain drives the system to grow two-dimensional [18], with a limited density of threading dislocations (TDs) and a flat surface [19].

Self-assembled QDs cannot be formed on {111} surfaces by means of the conventional Stranski–Krastanov (SK) mode due to the compressive strain-induced insertion of misfit dislocations at the substrate–epilayer interface [18,20]. However, the tensile strain-driven self-assembly of coherently strained III–V QDs on (111) surfaces was reported [21,22]. The droplet epitaxy (DE) technique [23,24,25] recently demonstrated the possibility to grow high-quality quantum nanostructures in lattice-matched and -mismatched systems with a high degree of control over the density, size, and shape of the nanostructures [26,27,28,29,30,31,32,33,34,35,36], rendering it suitable for the fabrication of single photon emitters and entangled photon sources [3,8,36,37]. The flexibility of DE is because the growth of III–V QDs is performed in two distinct steps. In the first, the Group III element is deposited on the substrate to form liquid droplets; in the second step, a flux of the Group V element is irradiated in order to crystallize the droplets in quantum nanostructures. As DE is not strain-driven, it can be exploited with a variety of material combinations and substrate orientations. Ha et al., and Tuktamyshev et al. recently reported the possibility to grow InAs QDs with DE on InAlAs MMBL deposited on singular [38] and vicinal [19] GaAs(111)A with a suitable single photon emission for conventional fiber communication in the C- and O-Band windows. InAs QDs emitting in the 1.3 μm band showed the expected high symmetry in the excitonic states, featuring an FFS of less than 20 μeV [19].

In this study, we investigate in more detail the growth of InAs QDs by DE on MMBL/GaAs(111)A vicinal misoriented substrates in order to identify the optimal QD fabrication process to obtain the QD emission at telecom bands. In particular, we concentrate our attention on strain relaxation in QDs and how it is affected by the capping procedure.

## 2. Materials and Methods

The samples studied in this work were grown on undoped semi-insulating GaAs(111)A substrates with a miscut of 2${}^{\circ }$ towards [$\bar{1}$$\bar{1}$ 2] in a solid-source MBE. The use of vicinal wafers allows for a high growth rate of thick epitaxial layers (e.g., distributed Bragg reflector (DBR)) without incurring the formation of triangular hillocks that is typical on singular (111) surfaces [39]. The use of the [$\bar{1}$$\bar{1}$ 2] direction of a miscut and its angle value are caused by the good growth performance shown in our previous works [37,39]; After a 85 nm GaAs buffer layer had grown at 520 °C with a growth rate of 0.5 ML/s, a 100 nm In${}_{0.6}$Al${}_{0.4}$As metamorphic barrier layer was deposited at 470 °C with the growth rate of 0.5 ML/s. Then, metallic indium was supplied with the growth rate of 0.01 ML/s at 370 °C to reach 1 equivalent ML (S1, S3, S3 and S4) and 0.15 equivalent ML (S5 and S6). During indium deposition, the background pressure was kept below $3\times 10^{-9}$ Torr. Then, an As${}_{4}$ flux was supplied for 8 min at the same temperature to crystallize the indium droplets into InAs QDs after the crystallization process In${}_{0.6}$Al${}_{0.4}$As capping layers (CLs) of different thickness had been deposited at 370 °C with the growth rate of 0.5 ML/s. The growth method relative to the samples for PL measurements is described elsewhere [19]. Metallic indium and CL thickness relative to all the samples presented in this study are summarized in Table 1.

The morphological characterization of the samples was performed ex situ with atomic force microscopy (AFM) in tapping mode using supersharp tips capable of a lateral resolution of about 2 nm. The numerical calculations of the emission energy of the quantum dots were performed using the envelope function approximation and an eight-band k·p model. The QDs were modeled as truncated pyramids with a triangular base with a fixed aspect ratio (AR) of 0.05, following the analysis of the AFM images. Further details of quantum calculations can be found elsewhere [19]. PL measurements were performed by exciting the sample with a 405 nm laser with a power density of ∼4.5 kW·cm${}^{-2}$. The sample was mounted in a closed-cycle cryostat and kept at a constant temperature of 15 K. The signal was filtered with a GaAs long-pass filter to remove light coming from the laser. The PL was acquired with a f/3.6 monochromator equipped with a 590 lines/mm grating with blaze at 1.3 μm. The detector was a cooled InGaAs photodetector (−15 °C). The signal-to-noise ratio was improved by using a lock-in amplifier.

## 3. Results

**Figure 1:**
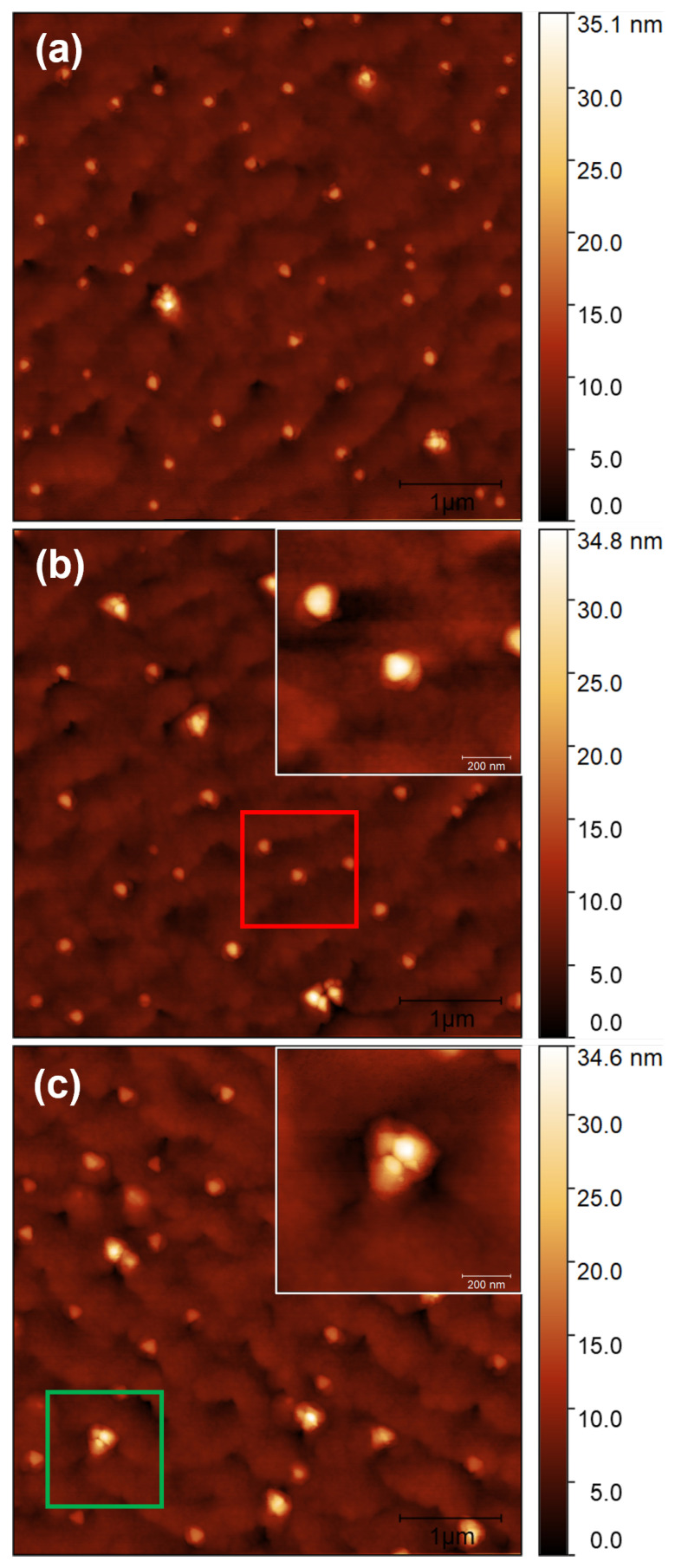
(**a**) A 5 × 5 μm${}^{2}$ AFM topography image of Sample S1 without any capping layer; (**b**) 5 × 5 μm${}^{2}$ AFM topography image of Sample S2 taken after the deposition of 5 nm of capping layer. (inset) A 1 × 1 μm${}^{2}$ AFM topography image relative to the red square in (**b**) Group A QDs with a truncated pyramidal shape; (**c**) 5 × 5 μm${}^{2}$ AFM topography image of Sample S3 taken after the deposition of 10 nm of capping layer. (inset) A 1 × 1 μm${}^{2}$ AFM topography image relative to the green square in (**c**) a Group B QD that was clearly dislocated.

Figure 1a shows the morphology of Sample S1 with uncapped self-assembled DE InAs QDs fabricated on a In${}_{0.6}$Al${}_{0.4}$As MMBL with the deposition of 1 ml of indium at 370 °C, followed by annealing in an As atmosphere at the same temperature. The average morphological characteristics of Sample S1 were measured on areas of 100 μm${}^{2}$ and are listed in Table 2. Details on the In${}_{0.6}$Al${}_{0.4}$As MMBL prior to QD growth can be found in our previous publication [19]. In${}_{0.6}$Al${}_{0.4}$As MMBL features a root mean square (RMS) roughness below 1 nm and a threading dislocation density (TDD) of the order of 1 × 10${}^{7}$ cm${}^{-2}$. Uncapped QDs on Sample S1 exhibited a truncated triangular pyramidal shape with an average height of 9.9 ± 3.4 nm and an average lateral size of 192 ± 60 nm. In this work, the lateral size was measured as the height of the triangular base of the QD. The density of the QDs was 2.1 × 10${}^{8}$ cm${}^{-2}$. The density of the QDs on this sample was comparable to the 2.5 × 10${}^{8}$ cm${}^{-2}$ density value reported in our previous publication [19]. The sample surface was populated by two distinct groups of QDs. The first and more numerous group (Group A) consisted of smaller QDs with heights ranging from 2 to 15 nm and a triangular flat top (an example is shown in the inset of Figure 1b). The second group (Group B) comprised larger QDs with a density of 2 × 10${}^{7}$ cm${}^{-2}$. These QDs exhibited heights of ≥15 nm and and irregular morphologies (an example is shown in the inset of Figure 1c).

All the QDs were surrounded by a two-dimensional structure. The formation of such a 2D layer could be attributed to the kinetically controlled diffusion of metal atoms out of the nanostructures during the crystallization step [36]. The material diffusion was not isotropic, but occurred preferentially along three equivalent <1$\bar{1}$0> directions on the (111) surface.

The optical properties of capped QDs can be predicted with quantum mechanical models on the basis of the morphology and composition of the uncapped nanostructures [32,40]. We calculated the expected emission wavelength from Group A QDs by means of a k · p approach, modeling the QD shape as a truncated pyramid with a fixed and very small aspect ratio ρ=0.05. Such a shape was derived from the actual QD shape measured on uncapped Sample S1 and it was in agreement with previous studies on InAs DE-QDs on InAlAs(111)A MMBL [38,41]. The simulation suggested a QD height of 2.4 and 3.6 nm for emissions at 1.3 and 1.55 μm, respectively (see Table 2). On the basis of numerical calculations, Group A of small InAs/In${}_{0.6}$Al${}_{0.4}$As QDs were expected to emit in telecom bands C and O with an extremely broad photoluminescence band.

**Figure 2:**
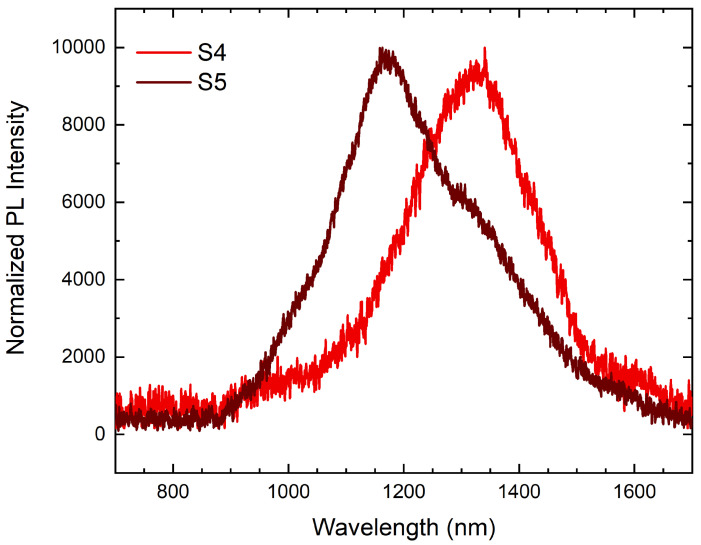
Normalized broadband PL spectrum measured at T = 15 K on Samples S4 (bright red, obtained by capping an identical sample to S1 (shown in Figure 1a) and S5 (dark red, obtained by capping an identical sample to S6 (shown in Appendix A, Figure A1), and excited with a 405 nm laser with power density of ∼4.5 kW·cm${}^{-2}$.

The photoluminescence (PL) spectrum measured after capping with 140 nm In${}_{0.6}$Al${}_{0.4}$As (Sample S4) is shown in Figure 2. The spectrum consisted of a broadband emission covering wavelengths from 1.10 to 1.50 μm. No emission was observed for wavelength in the C-Band window. This result differs from the results obtained from the numerical simulations. The observed behavior could have been the outcome of two possibly synergistic phenomena: (1) since QDs must be buried with a capping layer to act as an active optical layer, the capping procedure may have affected optically active QDs; (2) the low-threshold channel for plastic strain relaxation on {111}-oriented substrates may have introduced misfit dislocation at the QD–MMBL interface in the larger QDs, thus quenching their emission. The direct self-assembly of InAs QDs by on GaAs(111)A substrates by DE resulted in plastically relaxed islands [42].

As a matter of fact, it is widely reported that even a thin CL can modify the size, morphology, and surface density of InAs QDs [43,44,45,46,47,48,49]. The role played by Al-containing CL on the changes in the structure of the QDs and the recombination mechanisms is complicated. On one hand, the use of InAlAs as a strain-reducing layer (SRL) is widely reported to improve the emission intensity and the redshift of InAs QDs in the telecom band compared to other overlayers such as GaAs [47,50,51,52,53,54]. However, QDs embedded in an InAlAs matrix suffer from carrier hopping via defects, an Al-related nonradiative recombination center, and dislocation-related nonradiative channels. This deteriorates both PL intensity and exciton lifetime in the quantum dots compared to other embedding matrices (e.g., InGaAs) [55]. Liu et al. suggested that the formation of such defects is influenced by the low InAlAs growth temperature [56]. In order to shed some light on the observed difference between the simulation and the experimental measurements, we investigated the influence of the InAlAs CL on the morphology and hence the optical properties of InAs QDs grown by DE on a fully relaxed InAlAs MMBL. We used AFM surface characterization to analyze QDs buried under CLs of different thickness and compared them with surface QDs.

QDs on Sample S2 were covered with 5 nm of In${}_{0.6}$Al${}_{0.4}$As, whereas QDs on Sample S3 were covered with 10 nm of In${}_{0.6}$Al${}_{0.4}$As. Examples of AFM scans obtained on Samples S2 and S3 are shown in Figure 1b,c, respectively. The average morphological characteristics of Samples S2 and S3 were measured on areas of 100 μm${}^{2}$ and are listed in Table 3. Sample S2 was populated by the same two groups of QDs observed on Sample S1 with similar proportions. The total density of the QDs was comparable to the one measured on Sample S1. The total density on Sample S3 dropped by 38% compared to that of Sample S1. The QD density of Group B increased to 3.3 × 10${}^{7}$ cm${}^{-2}$.

AFM characterization also revealed that QDs maintained their triangular symmetry among all three samples (Figure 1). Sample S6 was obtained by depositing 0.15 ML of indium at 370 °C on a In${}_{0.6}$Al${}_{0.4}$As MMBL, followed by annealing in an As atmosphere at the same temperature. The average morphological characteristics of the surface dots on Sample S6 (see Appendix A, Figure A2) were measured on areas of 100 μm${}^{2}$ and are listed in Table 3. The photoluminescence spectrum measured after capping with 140 nm In${}_{0.6}$Al${}_{0.4}$As (Sample S5) is shown in Figure 2. The spectrum consisted of a broadband emission covering wavelengths from 1.10 to 1.50 μm, similar to what was observed on Sample S4.

## 4. Discussion

**Figure 3:**
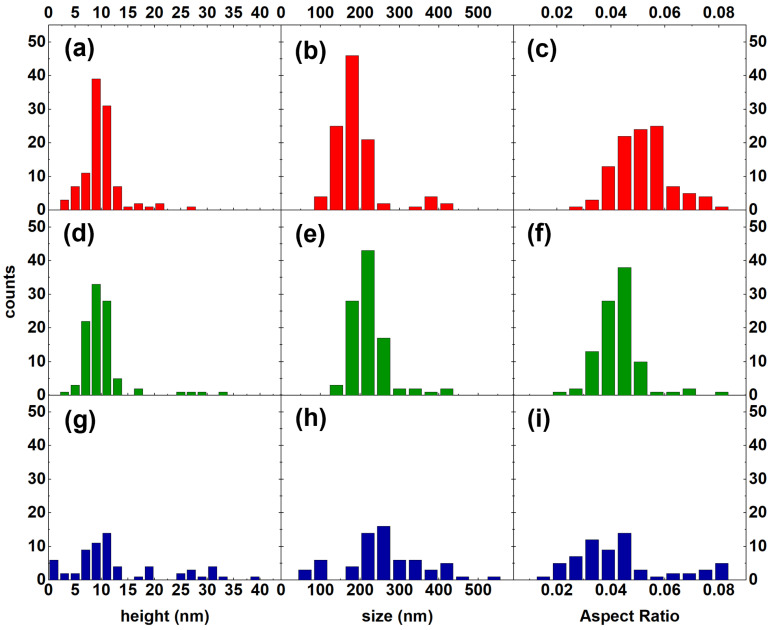
(**a**) Height, (**b**) lateral size, and (**c**) aspect ratio distributions relative to Sample S1 (no capping); (**d**) height, (**e**) lateral size, and (**f**) aspect ratio distributions relative to Sample S2 (5 nm capping); (**g**) height, (**h**) lateral size, and (**i**) aspect ratio distributions relative to Sample S3 (10 nm capping).

In order to investigate the effects of the InAlAs CL on the surface InAs QDs, we analyzed the changes in the morphology of the QDs among Samples S1, S2, and S3. The evolution of the QDs’ main features is shown in the histograms in Figure 3. The lateral size distribution exhibited a main peak that shifted toward higher values with increasing CL thickness (Figure 3b,e,h). From energetic considerations, it is expected that the InAlAs capping layer would preferentially grow on the fully relaxed InAlAs MMBL between the dots to reduce the surface curvature and minimize the surface energy. Hence, the top of the pseudomorphic InAs dots should not be covered by the InAlAs overlayer until the equivalent thickness of the total InAlAs deposition is at least equal to or larger than the dot height [57]. As a consequence of this, the height distribution should shift towards smaller values with increasing CL thickness.

However, the main height peak observed around 9–10 nm on Sample S1 (Figure 3a) did not exhibit any noticeable shift in Sample S2 (Figure 3d) or Sample S3 (Figure 3g). Furthermore, Sample S3 exhibited a noticeable increment of the dispersion of the height and the lateral size of the QDs (Figure 3g,h). Ferdos et al. observed that InAs dots covered by 1 ml of an Al-containing CL suffer from an initial height reduction, but further encapsulation does not influence the average height of dots and leads to wider height distributions instead [47]. We could assume that the drop in density can be attributed to the disappearance of smaller QDs that become completely buried by the overlayer. Partially buried QDs were accounted for the observed heights of ≤2 nm.

**Figure 4:**
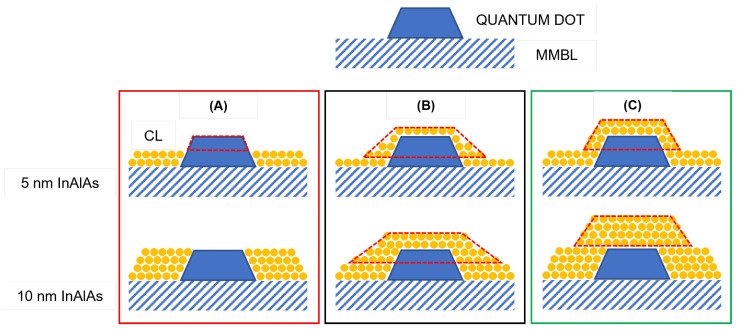
Graphical models of a QD covered by 5 and 10 nm CL (**A**) as if all the dots were pseudomorphic (PQD model); (**B**) as if material accumulations occurs on the dot due to a mass transfer mechanism; (**C**) as if the entire surface is covered by a CL of uniform thickness (CC model). The dashed red line marks the volume of the QDs that would be detected by AFM. (**A**) QD height decreases by an amount equal to the CL thickness. (**B**,**C**) QD height as seen by the AFM is constant.

In order to shed some light on why the height of the majority of the dots remains virtually unchanged, we measured the average area and volume occupied by one dot measured in Samples S1, S2 and S3. We then compared the measured values with the ones calculated (i) as if all the dots were ideally pseudomorphic and (ii) as if the entire surface was covered by a CL of uniform thickness, also known as conforming capping. Graphic representations of both scenarios are shown in Figure 4. In the pseudomorphic QD (PQD) model, the facets of the dots are buried by the CL, so that the QD height decreases by an amount equal to the CL thickness (Figure 4A). Area $A_{PQD}$ and volume $V_{PQD}$ occupied by a QD can be expressed as follows:

(1) $$A_{PQD}=\left({\left(h\times AR-2t/\tan\left(\alpha \right)\right)}^{2}/\sqrt{3}\right)$$

(2) $$V_{PQD}=\left(h-t\right)/3\times (A_{PQD}\times \left(1+\delta +\sqrt{\delta }\right)$$

In the conforming capping (CC) model, the QD is covered by a uniform layer such as a blanket; hence, its area $A_{CC}$ and volume $V_{CC}$ are increased (Figure 4C) according to:

(3) $$A_{CC}={\left(\left(h\times AR\right)+2t\tan\left(\alpha /2\right)\right)}^{2}/\sqrt{3})$$

(4) $$V_{CC}=\left(h\right)/3\times (A_{CC}\times \left(1+\delta +\sqrt{\delta }\right)$$

where *h* is the average height of the QDs, *t* is the CL thickness, α is the angle formed between one of the QD facets and the surface, and δ is the ratio between the two bases of the QDs.

**Figure 5:**
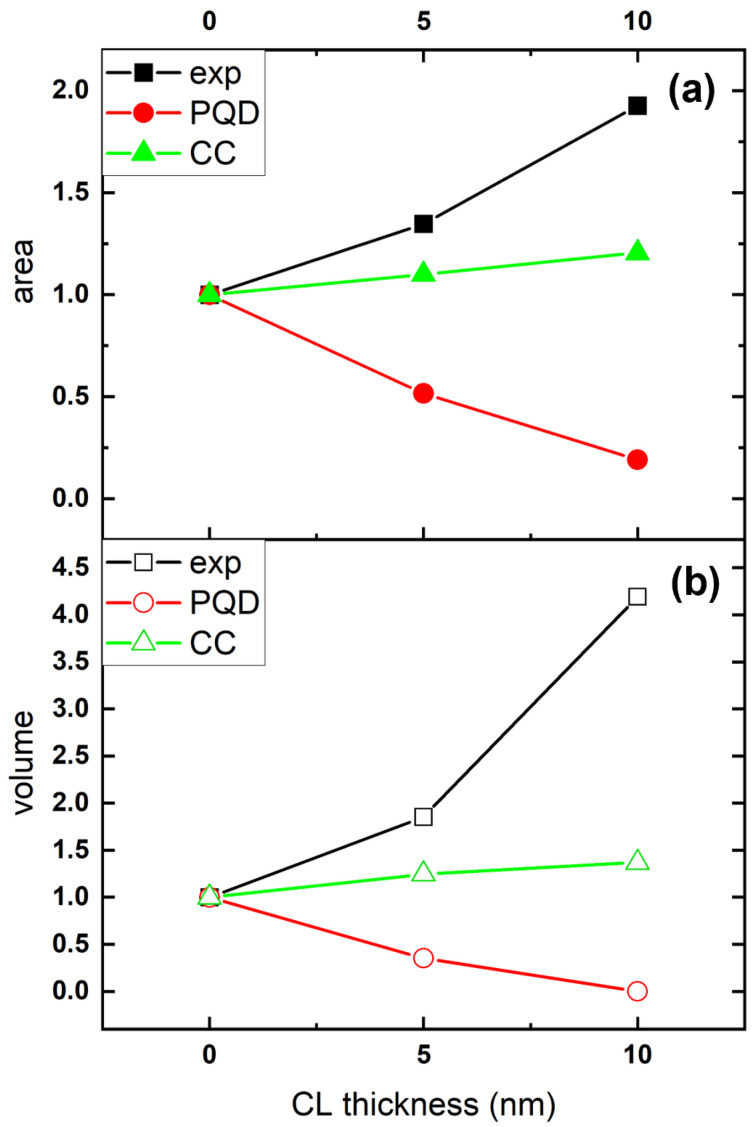
(**a**) Normalized average QD base area measured with AFM (black full squares), calculated with the PQD model (red full circles), and calculated with the CC model (green full triangles) as a function of the CL thickness; (**b**) normalized average QD volume measured with AFM (black hollow squares), calculated with the PQD model (red hollow circles), and calculated with the CC model (green hollow triangles) as a function of CL thickness.

Figure 5 shows the average values of area (Figure 5a) and volume (Figure 5b) occupied by one QD as a function of the CL thickness measured by AFM, and calculated according to the PQD (Equations (1) and (2)) and CC (Equations (3) and (4)) models shown in Figure 4A,C, respectively. The measured area and volume increased with the CL thickness up to ≈2 and ≈4 times the original values, respectively (black lines). On the one hand, in the pseudomorphic QD (PQD) model, both area and volume decreased to 0 as the QD became completely buried. On the other hand, the CC model clearly underestimated the real increment in area and volume measured with AFM. These results suggest that the simultaneous increment of both area and volume of the dot occurs as a consequence of the mass transfer towards the QD.

Al atoms accumulated on top of the QDs due to the species’ low mobility at the growth temperature. The accumulation of Al on the dots is widely reported to prevent In migration towards the surface [47,50,51,52,53] and hence the consequent dissolution of QDs usually occurring at temperatures of 370 °C or higher. Our results suggest that In atoms tend to diffuse towards the dots instead, and accumulate both on the top and especially at the perimeter of QDs. This causes a slight decrease in aspect ratio (AR) distribution, as shown in Figure 3c,f,i. The observed mass transfer can be explained by the presence of plastic relaxation of the strain due to the presence of dislocations within the QDs that were thicker than ≈2.6–2.7 nm. These plastically relaxed InAs QDs act like a sink for the more mobile species, which diffuse towards them to lower the elastic energy accumulated in the CL [58,59]. When an In${}_{x}$Al${}_{1-x}$As overlayer is deposited on a flat surface composed by adjacent areas of In${}_{x}$Al${}_{1-x}$As and InAs, the more mobile species (In) migrate towards the areas where the lattice mismatch between the In${}_{x}$Al${}_{1-x}$As overlayer and InAs is larger to minimize the total elastic energy accumulated in the overlayer. This results in a gradient in the composition and hence in the strain across the overlayer (e.g., In migrates to reduce the strong tensile strain above InAs and causes tensile strain above InAlAs). The mass transport increases with the increase in lattice mismatch between the overlayer and the substrate.

Several authors reported that InAs QDs can exhibit major morphological changes due to material redistribution occurring during the deposition of CLs of a few nanometers [43,49,57]. However, it is also possible that the plastic relaxation occurs during crystallization in all those dots that exceed the critical thickness above which insertion of misfit dislocation occurs. This critical thickness is a function of the system composition and the type of growth, 2- or 3-dimensional, and usually lies between 1.5 and 4 nm [60,61,62,63]. Ohtake et al. reported that, in the heteroepitaxy of InAs on singular GaAs(111)A, the InAs layer becomes plastically relaxed inplane due to the insertion of misfit dislocation above 0.39 nm InAs layer thickness [64]. A study by Chaldyshev et al. demonstrated that the critical relaxation size for QDs buried by a thick overgrowth is larger than the critical thickness for surface QDs [65].

**Figure A1:**
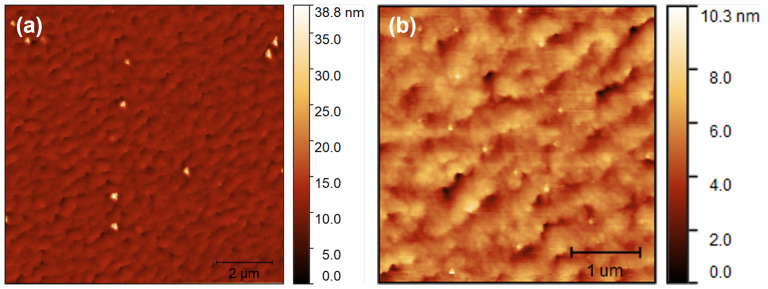
(**a**) 10 × 10 μm${}^{2}$ AFM topography image of Sample S6 without any capping layer; (**b**) 4 × 4 μm${}^{2}$ AFM topography image of sample S6.

**Figure A2:**
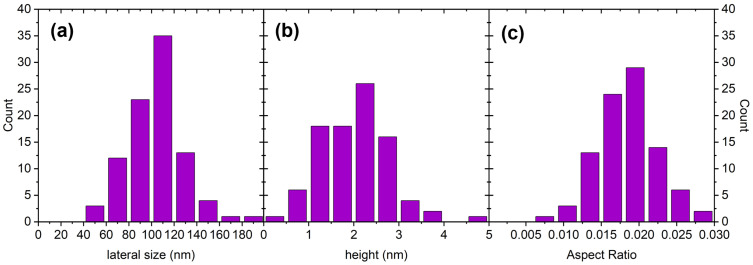
(**a**) Lateral size, (**b**) height and (**c**) aspect ratio distributions relative to Sample S6 (no capping).

**Figure A3:**
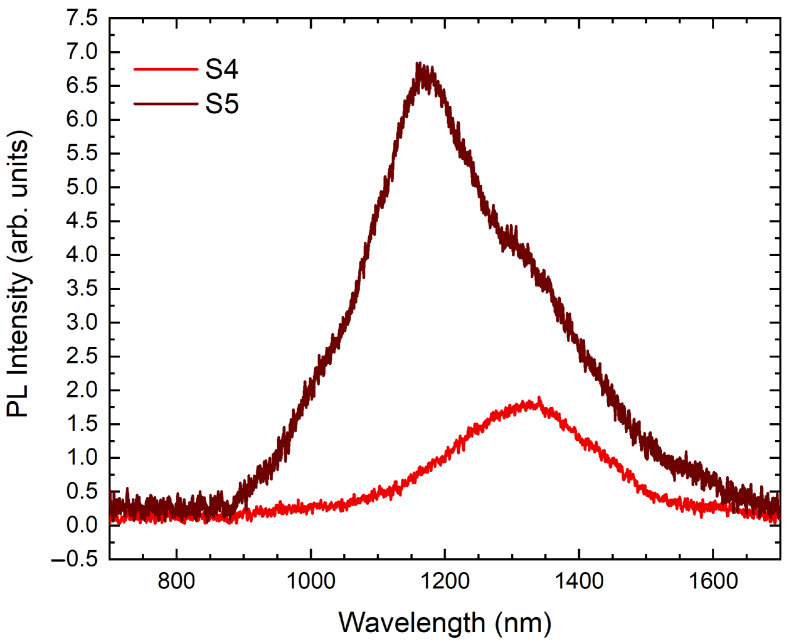
Absolute broadband PL spectrum measured at T = 15 K on Sample S4 (bright red, obtained by capping a sample identical to S1 (shown in Figure 1a) and Sample S5 (dark red, obtained by capping a sample identical to S6 (shown in Appendix A, Figure A1) and exciting with a 405 nm laser with power density of ∼4.5 kW·cm${}^{-2}$.

A possible alternative explanation of the observed blue shift (Figure 2) with respect to the expected emission (Table 2), calls for In/Al intermixing at the QD/barrier interface occurring during the deposition of the CL. In/Al intermixing can lead to an interdiffusion-driven blue shift of the QD emission with respect to the expected emission based on uncapped QDs. Such a blue shift must affect all QDs irrespective of their size. A size effect is expected anyway, with the blue shift increasing with the In interdiffusion length/QD size ratio. Sample S6 was grown by lowering the amount of deposited In to 0.15 ml in order to reduce the size of the QDs. QDs on Sample S6 exhibited an average height of 2.0 ± 0.7 nm (Table 3), with a large majority of QDs that were smaller than the critical thickness for plastic deformation. A detailed AFM characterization of Sample S6 can be found in Appendix A, Figure A1 and Figure A2. As shown in Figure 2 the PL spectrum measured after capping with In${}_{0.6}$Al${}_{0.4}$As (Sample S5) was in good agreement with our quantum calculations (Table 2) and covered the same emission range observed on S4. On the one hand, these results prove that the emission measured on Sample S4 came exclusively from dots smaller than ≈2.6–2.7 nm in height that were not plastically relaxed. On the other hand, these results exclude the occurrence of material intermixing among Ω QDs during the capping stage on Sample S5, as no blue shift of the emission was observed and hence on any other samples presented in this work. The absolute PL intensity measured on Sample S5, where ≈60% of the QDs were smaller than the critical thickness, was four times larger than the absolute intensity measured on Sample S4, where only ≈40% of the dots were optically active. This difference was in agreement with the ratio between the densities of pseudomorphic QDs populating Samples S5 and S4, which was ∼6. The spectra showing the absolute intensities measured on Samples S4 and S5 can be found in Figure A3 in Appendix A.

**Figure 6:**
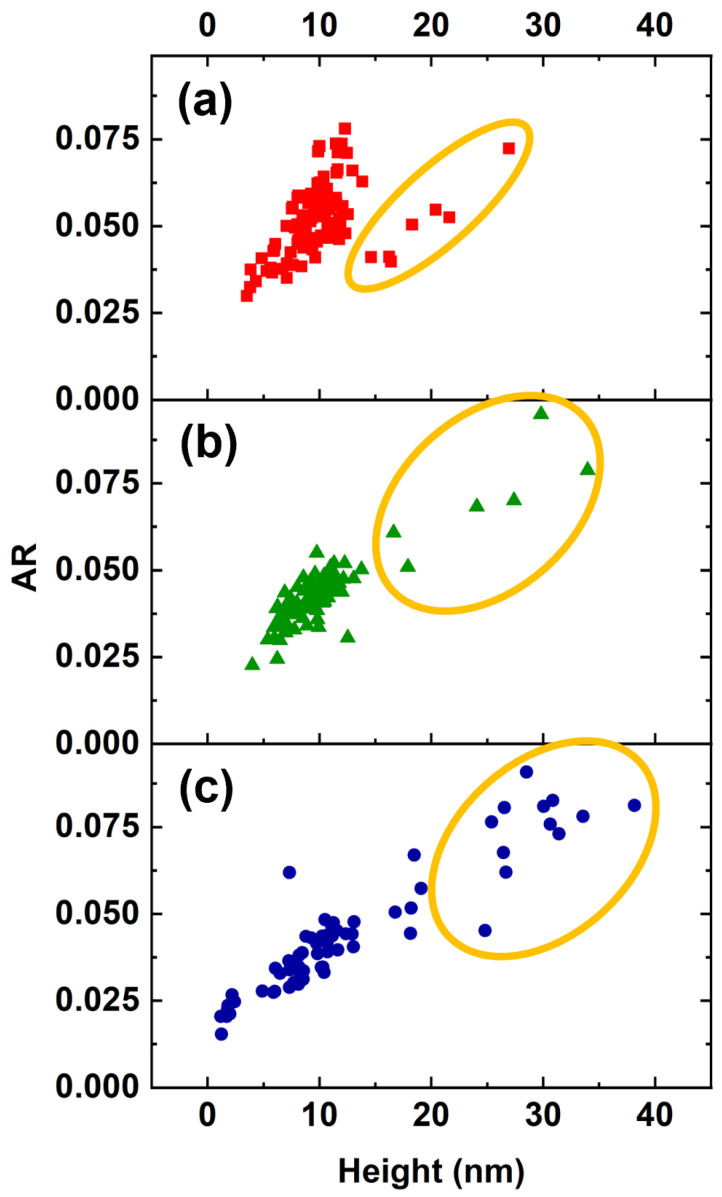
Aspect ratio as a function of the height measured on the single QDs for Sample S1 (**a**), Sample S2 (**b**), and Sample S3 (**c**). The data outlined in orange were measured on plastically relaxed Group B QDs. These QDs grew in density and height with increasing CL thickness.

We identified Group B as an island that formed with the droplet decoration of the TD arms intercepting the surface. Due to the preferential droplet nucleation at defects and the strain field induced by the TDs, these droplets were larger than the rest. Their irregular morphology (Figure 1c, inset) was due to the presence of a relevant plastic relaxation of the island strain. Their density was in agreement with the density of TDs in InAlAs MMBL on GaAs(111)A substrates [19]. The height of Group B dots increased with increasing CL thickness. In Sample S3, the total QD population was split into bimodal-type distribution (Figure 3g). Similar behavior was reported for InAs QDs capped with 10 nm AlAs layers [66]. AR distribution also exhibits a bimodal character, as shown in Figure 3i. When the AR of the QDs was plotted as a function of the height, it clearly showed two opposite behaviors (Figure 6): as the InAlAs CL thickness increased, the majority of the QDs were grouped around an average height value with a slight decrease in AR due to preferential material accumulation on their perimeter; Group B QDs (circled in orange) showed a tendency to grow vertically due to preferential material accumulation on their top. This behavior confirms the presence of misfit dislocations within Group B QDs as well. On the atomic surface, diffusion towards InAs QDs increased the average QD size above the critical threshold for the inset of misfit dislocations, which in turn resulted in the formation of large clusters [58,59]. However, our analysis did not allow for identifying the origin of the increment in Group B QDs, whether they developed from existing QDs or they formed on the surface during the CL deposition due to the inset of defects and new dislocations in the embedding InAlAs matrix.

## 5. Conclusions

In conclusion, DE InAs/InAlAs QDs on vicinal Ga(111)A that were expected to emit in the telecom C and O bands exhibited a broadband PL with spectral weight limited at 1.50 μm. Our AFM characterization revealed material accumulation on more than 60% of the QDs during the CL deposition up to 10 nm. We attribute this mass transfer to the insertion of strain-induced misfit dislocation within the QDs that exceeded a critical thickness of 2.5 nm. QDs that were plastically relaxed acted as nonradiative recombination centers and did not contribute to the measured PL spectrum. Only the smallest QDs retained their coherence and were accounted for in the observed emission in the telecom O-band.

## Figures and Tables

**Table 1:** Amounts of In and CL thickness relative to all the samples presented in this study (S1–S6).

Sample	In [ML]	CL [nm]
S1	1.0	0
S2	1.0	5
S3	1.0	10
S4	1.0	140
S5	0.15	0
S6	0.15	140

**Table 2:** Simulated optimal size of a InAs/InAlAs QD with a fixed AR of 0.05.

Wavelength [nm]	Height [nm]
1300	2.4
1550	3.6

**Table 3:** Average morphological features of QDs grown with different In MLs measured with AFM on the samples covered by CLs of different thickness.

Sample	In (ML)	CL (nm)	QD Density (cm^−2^)	QD Height (nm)	QD Lateral Size (nm)	QDs AR
S1	1.0	0	2.10 ± 0.20 × 10${}^{8}$	9.9 ± 3.4	191.8 ± 60.1	0.052 ± 0.019
S2	1.0	5	1.96 ± 0.20 × 10${}^{8}$	10.1 ± 4.5	228.2 ± 48.5	0.043 ± 0.009
S3	1.0	10	1.30 ± 0.20 × 10${}^{8}$	12.8 ± 9.1	260.0 ± 101.8	0.044 ± 0.028
S6	0.15	0	9.20 ± 0.20 × 10${}^{8}$	2.0 ± 0.7	106.4 ± 23.6	0.018 ± 0.004

## Data Availability

Not applicable.

## Abbreviations

The following abbreviations are used in this manuscript:

MBE	Molecular Beam Epitaxy
QD	Quantum Dot
MMBL	MetaMorphic Buffer Layer
CL	Capping Layer
PL	PhotoLuminescence
TD	Threading Dislocations
SK	Stransky–Krastanov
DE	Droplet Epitaxy
PQD	Pseudomorphic Quantum Dots
CC	Conforming Capping
